# The Australian clinical trial landscape: Perceptions of rural, regional and remote health service capacity and capability

**DOI:** 10.1186/s12961-024-01270-z

**Published:** 2024-12-19

**Authors:** Jenna Graffini, Karen Johnston, Alison Farrington, Steven M. McPhail, Sarah Larkins

**Affiliations:** 1 College of Medicine and Dentistry, James Cook University, Townsville, QLD Australia; 2 Australian Institute of Tropical Health and Medicine, James Cook University, Townsville, QLD Australia; 3 Australian Centre for Health Services Innovation and Centre for Healthcare Transformation, School of Public Health and Social Work, Faculty of Health, Queensland University of Technology, Brisbane, QLD Australia

**Keywords:** Teletrials, Clinical trials, Rural, regional and remote health, Health services

## Abstract

**Background:**

Access to clinical trials is limited for rural, regional and remote Australians, adding to the current health inequity between rural and metropolitan populations. The Australasian Teletrial Model was developed to bring clinical trials “closer to home”. In 2020, the Australian Teletrial Program was funded to expand and support the uptake of the model across six Australian states and territories. The aim of this study was to explore and describe the clinical trial landscape in Australia prior to the implementation of the Australian Teletrial Program with a particular focus on rural, regional and remote health services.

**Methods:**

This qualitative study provides a descriptive exploration of the clinical trial landscape across rural, regional and remote Australia. Data were obtained from semi-structured interviews (*n* = 33) and one focus group (*n* = 5) involving clinical trial stakeholders between August 2022 and May 2023. Deductive then inductive thematic analysis used the broad topic areas of the interview schedule as a framework, as follows: education and training, workforce, equipment and services, clinical trial sites, participant recruitment and clinical trial approval process.

**Results:**

This study identified barriers that are generalizable to the Australian clinical trial landscape and those specific to the rural, regional and remote health service context. The main barriers to conducting clinical trials in rural, regional and remote areas were lack of investment and engagement on the part of health service executives, workforce limitations, inconsistent training, lack of physical infrastructure and competing clinical priorities. Despite these challenges, clinicians reported enthusiasm for conducting clinical trials, and opportunities were reported for these health services to partner with larger metropolitan/regional health services, regional universities and communities to support the growth of clinical trial capability and capacity.

**Conclusions:**

The clinical trial landscape in Australian health services varies in terms of quality and availability of training, workforce capacity, executive support, site capability and approval processes. The Australian Teletrial Program has an immense opportunity to overcome some of the reported challenges by supporting capacity and capability building. Ultimately, however, sustainable reform to bring trials closer to home requires a collaborative approach that considers implementation strategies across all levels of the health service and government, alongside other initiatives.

**Supplementary Information:**

The online version contains supplementary material available at 10.1186/s12961-024-01270-z.

## Background

Systematic reviews and meta-analysis of good-quality randomized controlled trials are considered to provide the highest level of evidence of health intervention effectiveness [1]. Not only do clinical trials contribute to evidence-based healthcare delivery, they also provide benefits directly to participants through comprehensive health screening, access to new interventions and enhanced clinical care [2]. Despite the known benefits, many people living in rural, regional and remote (RRR) areas are unable to take part in a clinical trial, in most part due to their geographical remoteness from trial centres, requiring considerable travel time and expenses [3]. In 2022, 28% of the Australian population lived outside of major cities, in RRR areas, with the burden of disease increasing with greater remoteness [4]. Rurality is defined by the Modified Monash Model (MMM) classification system, which measures geographical remoteness and population size on a scale from MMM1 (major city) to MMM7 (very remote)[5]. Areas classified as MMM2–MMM7 are considered RRR. While it is not known what proportion of Australian clinical trial participants live in RRR areas, people living in these areas have an interest in and a perceived importance of having access to clinical trials [6–8]. In addition, a study by Lockery et al. [9] found that general practitioners in regional Australia were 45% more likely to recruit a trial participant than metropolitan general practitioners. Therefore, there is an interest and need for clinical trials, but concerted efforts are required for people living in these regions to overcome barriers to participation.

The Australasian Teletrial Model (ATM), inspired by the telehealth and the tele-oncology model, was developed by the Clinical Oncology Society of Australia to make clinical trials more accessible to people living in RRR Australia [10]. The ATM can be conceptualized as a hub-and-spoke (cluster) arrangement with a lead (primary) site overseeing other sites that are typically smaller (satellite sites) facilitated by a supervision plan and sub-contractual agreements [11]. The model was successfully piloted between 2017 and 2020 in three jurisdictions, and a national compendium was released in 2021 to guide the use of the ATM [12–14]. Federal funding was subsequently awarded for critical infrastructure and coordination of the Australian Teletrial Program (ATP), which uses the ATM with the intention of allowing people living in RRR areas to access trials closer to home [15]. As part of the program a Regional Central Coordinating Centre (RCCC) has been established in each participating state and territory (Queensland, Northern Territory, Western Australia, South Australia, Victoria and Tasmania) and tasked with implementing the program within their jurisdiction. New South Wales and Australian Capital Territory are the only Australian state and territory not part of the ATP, but are separately implementing measures with shared vision to broaden access to clinical trials [15]. Considering current and future investment to advance access to clinical trials for people living in RRR areas, it is important to understand the current clinical trial landscape across all of Australia as well as for RRR specifically. This includes identifying potential barriers and enablers to successful implementation of the ATP [16]. This manuscript outlines the experiences, opinions and perceptions held by clinical trial stakeholders about the clinical trial landscape in Australia as part of a baseline assessment at ATP commencement, with a particular focus on understanding existing capacity and capability in RRR health services.

## Methods

Context mapping of the Australian clinical trial landscape was performed using a convergent mixed method approach that involved a desktop analysis, environmental scan and stakeholder interviews. This manuscript reports the findings from in-depth semi-structured interviews with stakeholders across the Australian clinical trial landscape.

### Setting

This Australian national study focused on public hospital and health services in metropolitan and RRR locations across the states and territories participating in the ATP. These states and territories deliver healthcare through a combination of statewide health services and individually governed geographically defined health regions, variously referred to as “local health networks”, “hospital and health services” or “health services”. Here, these will be collectively referred to as “health services”. Each of the health services are governed by a board of directors and executive officers and influenced by the health profile and demands of the local community as well as the relevant federal and state and territory legislation, policies and the Australian Commission on Safety and Quality in Healthcare [17, 18]. In many RRR areas, health services are also responsible for providing primary care to the local community although the focus of this study is the public hospital sector. The heterogeneous nature and structure of health services is an important contextual factor for the ATP and the interpretation of the study findings.

### Participants and recruitment

Participants were recruited from three groups of clinical trial stakeholders: clinician researchers (*n* = 8), health service stakeholders (*n* = 17) and industry stakeholders (*n* = 13). Clinician researchers included healthcare providers (doctors, nurses and allied health professionals) and clinical trial professionals (trial managers, pharmacists, coordinators, administrators and nurses) involved in the direct coordination, management, administration, development or delivery of clinical trials at a health service. Health service stakeholders included staff working within health services or departments of health that support research activity but do not directly conduct trials, such as directors of research, research governance officers, executive officers and RCCC staff. Industry stakeholders included commercial sponsors, academic researchers from universities, managers and directors from research institutes, research collaboratives/networks and clinical trial organizations.

Due to the specific topic of this research, a combination of convenience (direct invitation to ATP stakeholders), snowball (one participant invites another participant) and purposive sampling of participants was used to ensure that a range of experiences and locations were represented [19]. Clinician researchers and health service stakeholders were identified with the assistance of the RCCCs. Industry stakeholders were identified through professional networks and advisory groups, snowballing and internet searching. Identified stakeholders were emailed an invitation by the research team or RCCC. Invited stakeholders contacted the research team if they were interested. Recruitment continued until representation of at least one clinician researcher and one health service clinical trial stakeholder from each jurisdiction was achieved. At this stage, limited new ideas were emerging in the interviews.

### Data collection

A semi-structured interview guide was used, allowing flexibility to shape questions around the stakeholder’s background and explore concepts. The interview guide contained open-ended questions on the following topics: education and training, workforce, equipment and services, recruitment and policy and processes (Additional file 1). Interviews were conducted by a senior research officer with 10 years’ experience working in the clinical trial sector within a regional tertiary teaching hospital and university. Participant informed consent was collected by email prior to the scheduled interview. Consent for audio-recording and publication was confirmed verbally at the time of the interview. Interviews were audio-recorded and transcribed using transcription software (Otter version 3.14 and NVIVO release version 1.7). Transcriptions were de-identified and checked for accuracy by an experienced qualitative research officer. Transcripts were imported to NVIVO for qualitative analysis.

### Data analysis

Transcripts underwent initial coding by two researchers and were deductively clustered according to the question topic. An iterative thematic coding approach [20] was then used to inductively create and analyse codes, grouping them into themes for each interview topic. To differentiate themes between RRR and the general Australian clinical trial landscape, themes were categorized to the general context if they were discussed by all participants or if frequently reported by only metropolitan-based participants. Themes were categorized to the RRR context if they were mainly discussed by participants from RRR settings. Codes, themes and concepts were refined over time as subsequent interviews were undertaken. This process was iterative, and three researchers (J.G., K.J. and S.L.) met regularly to discuss concepts that arose during the analysis.

### Ethics

This study obtained human research ethics approval from the Townsville Hospital and Health Service (HREC/2022/QTHS/85173), and reciprocal approval from Northern Territory Health (2022–4389), Queensland University of Technology (5963) and James Cook University (H8829). Research governance approvals were obtained, where necessary, from participating health services.

## Results

A total of 38 stakeholders participated in interviews (*n* = 33) or focus groups (*n* = 5) between August 2022 and May 2023 (Table 1). Interviews were conducted in-person or across an online videoconferencing platform. Interviews ranged from 35 to 127 min in duration with a mean of 62 min. The MMM classification [5] was used to classify rurality of participants’ locations; 21 were based in MMM 2–7 regions (RRR), and 17 were based in MMM 1 regions (metropolitan). Participant experience in clinical trials ranged from 4 to 30 years.

**Table 1:** Demographic characteristics of participants

	*N* (%)
*Total number of participants*	38
Gender, female	26 (68%)
Years’ experience, median (range)	14 (4–30)
Located in RRR region (MMM2–7)	21 (55%)
Located in a metropolitan region (MMM1)	17 (45%)
*Type of participant*	
Clinician researcher	8 (21%)
Health service	17 (45%)
Industry	13 (34%)
University	*5*
Research institute/collaborative	*5*
Commercial	*3*
*State or territory*	
Queensland	11 (29%)^*^
Northern Territory	7 (18%)
Western Australia	4 (11%)
Victoria	5 (13%)
South Australia	4 (11%)
Tasmania	2 (5%)
National^**^	5 (13%)

^*^Five QLD participants were a part of one focus group

^**^Stakeholders who were experienced and positioned across multiple jurisdictions

The major themes and sub-themes that emerged under each question topic are tabulated in Table 2 and further elaborated on in this section. Further supporting quotes for each sub-theme can be found in Supplementary File 2. As the aim of the ATP is to increase clinical trial access in RRR regions, the themes arising within the RRR context have been prioritized in this section.

**Table 2:** Major themes and sub-themes arising from interviews

Interview topic	Themes ▪ Sub-themes
Education and training	Variation in clinical trial training received with GCP the minimum standard. ▪ On-the-job training was reported to be the most common form of training. ▪ Learning environment for training depended on types of trials and the people in the environment. ▪ GCP training and study protocol training were reported to be the minimum standard training required for site trial staff.
	Clinical trial training is relative to clinical trial activity, and thus limited in RRR. ▪ RRR health services without active trials did not have exposure to or need for staff training. ▪ More sharing of knowledge, mentoring and training is needed in RRR health services where clinical trial activity is developing.
Workforce	Trial workforce challenges are complex, persistent and across the board. ▪ Competitive funding models contributes to insecure and fractional work for trial staff. ▪ Lack of professional recognition and career progression affects the attraction and retention of clinical trial staff. ▪ Clinician researchers feel under-supported due to increasing clinical workloads and lack of protected and remunerated research time. ▪ More support is needed to stabilize the clinical trial workforce.
	Unique workforce challenges in RRR health services exist alongside an appetite for trials. ▪ Low to no clinical trial workforce in RRR health services with variation across departments. ▪ Severe workforce shortages risks destabilizing clinical trial capacity in RRR health services. ▪ Call for trial staff to be consolidated across whole of RRR health services. ▪ RRR clinicians reported being passionate about providing their patients access to a clinical trial.
Equipment and services	Metropolitan sites generally have access to more resources to conduct clinical trials. ▪ Well-established health services that have a partnership with research institutes are better resourced to run clinical trials. ▪ Unpartnered health services or siloed research groups are likely to face challenges of sharing equipment and services with routine clinical care. ▪ Unexplored issues with clinical trial digital infrastructure.
	By contrast, RRR health services are insufficiently resourced for clinical trials. ▪ Lack of physical space, specialized services and capacity of services limited clinical trial capacity in RRR health services. The degree of limitation increased with remoteness. ▪ Regional university infrastructure might provide opportunity for providing space.
Clinical trial sites	An enabling culture, capacity and system is essential to running a successful site. ▪ Well-established clinical trial units are currently funded by a combination of grants and commercial sponsored funding. ▪ Understanding clinical trial business is important for all health services wanting a sustainable and financially stable trial site. ▪ Research culture of the health service is essential to a productive and sustainable clinical trial site. ▪ Investment, engagement and support by executives/directors that aligns with a well-considered research strategy is needed.
	Highly variable clinical trial capacity and capability across RRR regions. ▪ Low to no levels of clinical trial activity outside capital cities, particularly in NT, WA, SA and TAS. ▪ Frustration felt by RRR clinicians who are not able to provide trials to their patients due to lack of site capacity. ▪ Growing optimism and momentum for more equitable access to clinical trials at RRR sites.
Participant recruitment	Always challenging, but work is being done to break down the barriers. ▪ Streamlined and efficient processes to identify, recruit and involve participants is important to successful recruitment. ▪ General challenges to recruitment include participant burden, strict criteria, attitudes towards trials and language and cultural barriers. ▪ Thorough consumer engagement, careful protocol planning, monitoring risks and adapting when needed were identified as key elements to prevent recruitment challenges.
	Unique challenges in RRR health services but retention might be better than metropolitan sites. ▪ RRR clinical trial participants travel vast distances to take part. ▪ Clinical trial participation of RRR patients in cities is impractical and disruptive to care. ▪ Recruitment and retention of participants in RRR areas might be equivalent or better than cities. ▪ Cultural factors need to be taken into consideration.
Clinical trial approval processes	The approval process is long and variable across Australia. ▪ Significant reforms underway in Australia at state and national levels that aim to improve the capacity and capability of health services to conduct high-quality clinical trials. ▪ Variation in research governance approval process between health services and across jurisdictions. ▪ Long delays are reported throughout the research governance process. ▪ Conflicting expectations between health service research governance and clinician researchers.
	Lack of clinical trial maturity is an additional challenge for RRR health services. ▪ Potential for ATM to support RRR health services.

GCP, Good Clinical Practice; NT, Northern Territory; WA, West Australia; SA, South Australia; TAS, Tasmania

### Clinical trial education and training

#### Variation in clinical trial training received, with good clinical practice (GCP) being the minimum standard

Skilled and experienced clinical trial teams were reported to bring many benefits, including safer trials, quality data, smoother site set-up and faster participant recruitment rates. Historically, clinical trial training has primarily been “learn on the job” (Industry 5), where training, if available, was provided by study sponsor organizations and experienced staff and driven by clinical trial standards set by regulatory bodies. Participants reported that this resulted in an uneven distribution of skilled trial staff across commercial and investigator-lead clinical trial sectors. Mandatory clinical trial training for trial staff and investigators was reported to involve Good Clinical Practice (GCP) training, offered through a variety of modes and viewed by participants as minimal and insufficient. Participants, particularly from the industry sector, consistently discussed the need to increase the standard of clinical trial training to ensure all trial staff and investigators are trained to meet relevant technical and role-specific competencies.

#### Clinical trial activity in RRR limits the availability of training

Skilled clinical trial staff were most likely to exist in metropolitan or large regional tertiary hospitals rather than in smaller RRR areas, particularly in oncology, where higher levels of clinical trial activity existed. It was perceived that RRR health services without active clinical trials did not have exposure to learning environments or any reason to undertake clinical trial training:

Because why would you send someone for pilot training when they’re not going to fly the plane? You show them the plane. They will train. They will learn how to fly. But if there’s no plane who is going to go for a pilot training? (Clinician Researcher 2)

Pockets of trial activity, largely in oncology, were reported in some regional health services; these provided subsequent training, mentorship and capacity-building programs. In regional Victoria, several oncology clinical trial training and capacity-building initiatives existed, including the Regional Victorian Trials Alliance, Equity (ReViTALISE) Program, Alfred Health Trial Hub partnership program and the Victorian Comprehensive Cancer Centre (VCCC) SKILLED Clinical Trial Internship program. In Queensland, several large regional tertiary hospitals with clinical trial activity were reported to provide clinical trial training and general research skills workshops along with training and education partnerships with universities. Many participants expressed a need for a sharing of knowledge, mentoring and training with RRR health services where clinical trial activity is developing to enhance trial workforce capability.

### Workforce

#### Trial workforce challenges are complex, persistent and widespread.

Insecure work, characterized by fractional appointments and short-term contracts, was reported to be a major challenge in attracting and retaining skilled staff and a consequence of the way most hospital departments were funded to conduct trials (an inconsistent stream of grants or industry-sponsored payments). Securing enough funding to employ clinical trial professionals on an ongoing basis, particularly for investigator-initiated trials, was reportedly a significant and ongoing challenge. Details of the funding challenges are further discussed in the Clinical Trial Site section.

Participants also reported a lack of professional recognition of clinical trial professional roles (such as clinical trial coordinators, managers and nurses) on the part of health services, demonstrated by inconsistent position descriptions and pay levels, and lack of training and career progression pathways. Participants argued professional recognition and employment stability are critical enablers in attracting and retaining staff. Absence of these conditions was reported to have contributed to a chronic shortage of skilled clinical trial professionals and a phenomenon of clinical trial professionals moving out of health services into industry roles where they received higher pay, career progression pathways and more secure contracts.

Finally, competing pressures placed on the general workforce across health services were reported as an ongoing challenge. For clinical trial professionals, the coronavirus disease 2019 (COVID-19) response had disrupted and left the workforce exhausted. For clinician-investigators, performing their clinical trial duties as an in-kind contribution on top of their clinical workload was increasingly difficult. While many clinicians reported a passion for and interest in clinical trials, some felt the role had become unmanageable and unappealing because of increasing clinical workload, administrative burden, funding challenges and lack of tangible support.

Most participants expressed that health service executives need to do more to stabilize the clinical trial workforce by investing in clinical trial professional positions and remunerated research time for clinician-investigators*.*

#### Unique workforce challenges in RRR health services exist alongside an appetite for trials

Generally, the clinical trial workforce in RRR health services was reported as low or not existent, relative to clinical trial activity. In the majority of RRR health services that have clinical trial professionals, the staff were either spread out or concentrated in siloed departments.

The clinical trial workforce in RRR health services was largely perceived to reflect the general healthcare workforce, described as limited and consistently changing due to short-term clinical training programs, lack of medical specialist positions and lifestyle, social and seasonal factors. It was reportedly not unusual for staff to work autonomously, in isolation, in multiple roles or across departments, at times without leave cover. The inherent high turnover of the RRR health service workforce was reported to destabilize clinical trial capacity, with one clinician researcher in a remote hospital reporting having to absorb the workload of clinical trial staff member who had left when the positioned remained unfilled:

[…] The screening, recruitment, consenting, data collection, data entry filter, fell on me to do, which as a clinician with all those other things has become unsustainable. […] I find myself some 6 months behind in data entry. (Clinician Researcher 8)

To address some of the workforce issues, participants working in RRR health services suggested that local staff could be upskilled, and existing clinical trial professionals could be consolidated to provide support and mentorship across the whole health service. Despite the challenges, RRR clinician researchers consistently reported interest, motivation and passion to provide their patients with access to a clinical trial.

### Equipment and services

#### Metropolitan sites generally have access to more resources to conduct clinical trials

Well-established hospital clinical trial units that had partnerships with neighbouring or integrated research institutes were reported to have effective systems and processes established to use, share and continuously fund research-specific equipment and services that bypass the need to use public hospital equipment and services. Health services without these established partnerships were reported to compete with the health service’s clinical demands for space, equipment and services, thus impacting their ability to carry out the trial. While equipment and services for clinical trials were an obvious consideration for clinical trials, digital infrastructure such as trial software and online platforms was reported by one health service participant to be an unexplored essential consideration, with multiple software programs being used for similar functions, creating duplication and inefficiencies for sites.

#### RRR health services are typically insufficiently resourced for clinical trials

Availability of basic equipment and services to conduct clinical trials in RRR health services was dependent on the size of the health services and their experience in conducting clinical trials. Although most large regional hospitals were reported to have access to most basic equipment (for example, vital sign and anthropometric devices) and services required to conduct clinical trials (for example, pathology, medical imaging and pharmacy), there were reported issues of lack of physical space, specialized services (for example, aseptic manufacturing, specialized pathology and histology tests) and competition for services that were already at full capacity with day-to-day clinical demands.

We got a lot of pushbacks from pathology, radiology […] if it’s not a standard care, if it’s for clinical trials, then they might say no, because [they’re at] full capacity. (Health Service 3)

Several regional health services were reported to routinely outsource day-to-day clinical services such as medical imaging [particularly computed tomography (CT) and positron-emission tomography (PET) scans], pathology and aseptic manufacturing. While this was reported to have added a layer of complexity around the coordination of clinical trials across multiple facilities, some stakeholders acknowledged that outsourcing tests to private providers was more streamlined and reliable than using the in-house services: “It just sometimes can be hit and miss with the hospital’s capacity to do [imaging].” (Health Service 9).

Rural and remote health services were reported to be most limited in services needed for clinical trials, due to distance and additional logistics required for transportation and storage of drugs and blood samples. These services reportedly struggled to resource basic clinical trial equipment such as computers and find space for storage for trial drugs and site files, desk space and even freezer space:

They (rural health service) have […] four cupboards for pharmacy across [four MMM7 hospitals] and they can’t fit anything [for] clinical trials. So, if you wanted to buy them a minus 80 freezer, they got no space, it’s got to go outside. (Health Service 4)

Pathology units and services in some rural and remote health services were reported to be “very basic” (Clinician Researcher 3) and “do everything manually” (Health Service 3) with lack of capacity or ability to perform certain tests due to either lack of or out-of-date equipment requiring more manual and time-consuming processing. Despite these challenges one participant recognized opportunities to partner with regional universities with rural and regional training hubs who could provide infrastructure, resources and space.

### Clinical trial sites

#### An enabling culture, funding and capacity is essential

The ideal clinical trial site was categorized by participants to have an enabling environment; experienced and engaged team; efficient research processes; and appropriate physical capacity and infrastructure (Table 3). Funding issues, inconsistent site capacity and research leadership and lack of organizational research culture were the main reported challenges to conducting clinical trials.

**Table 3:** Reported characteristics of the ideal clinical trial site

Characteristic theme	Characteristics sub-theme	Example quotes
Enabling environment	▪ Positive research culture ▪ Executive support and investment ▪ Research a priority in strategic plan ▪ Supportive and knowledgeable departments ▪ Embedded research into core services	*“You need a culture that’s supportive of clinical trials. […] it can be either the culture within the unit, all the way up to, you know, having research as a strategic game of the bigger organization […] you know, people setting the direction, handing out the resources or agreeing that clinical trials are really important and that then links up all the way down to the specialty, the specialist teams.” (Health Service 14)*
Experienced and engaged team	▪ Multidisciplinary team ▪ Trained, experienced and engaged staff ▪ PI with interest, experience and capacity	*“A champion PI, who is invested in the trial, who sees trials and research at his site as a business to offer patients better care or different care or what additional care. They’ve got to see it as a business because they have to pay their staff out of the research budget.” (Industry 3)*
Efficient research processes	▪ High-quality data ▪ Quick participant recruitment ▪ Efficient processes ▪ Minimal errors	*“For investigator-initiated trials you need to be efficient because you are, you know, working off the smell of an oily rag or you need to be efficient because the way that you’re going to continue to attract commercially sponsored trials is by offering value for money.” (Industry 14)*
Appropriate physical capacity	▪ Calibrated equipment readily available ▪ Access to services with capacity ▪ Physical space ▪ IT and software infrastructure	*“We have a clinical research facility here with everything that we would need, so, you know, independent rooms, equipment, medical imaging. We have reception, we have waiting rooms and, you know, we’re not improvising.” (Industry 12)*

PI, principal investigator

Lack of stable funding was one of the greatest reported threats to clinical trials. Participants emphasized that research grants did not cover the full costs of investigator-initiated clinical trials, particularly salaries, due to the restrictive personnel salary support packages and perceived declining research grant success rate from significant competitive funders such as National Health and Medical Research Council and the Medical Research Future Fund. Participants from well-established clinical trial units reported revenue from grants and a steady stream of commercially sponsored trials, which when well managed were sustainable and had resources to support investigator-lead trials. It was reported that sites needed capacity, experience, reputation and business acumen to keep an incoming pipeline of trials and consistent revenue streams. However, for small teams, inexperienced sites or fields unlikely to attract industry sponsorship, maintaining consistent revenue is significantly challenging and ultimately impacts the long-term clinical trial capacity of the site.

Participants frequently reported inconsistent clinical trial capacity across both metropolitan and regional health services. Capacity was described as existing in “pockets of excellence” (Health Service 14) and “siloed” (Health Service 11) departments that were often driven by individual well-meaning and enthusiastic clinicians, referred to as “champions” (Industry 3). Clinicians' level of experience and clinical trial knowledge to conduct clinical trials was reported to vary, with some clinicians not understanding the clinical trial process or requirements for a trial within a health system. This was particularly the case for clinicians who did not have support from trial coordinators or managers to assist in administrative processes such as governance applications, budget and contractual negotiations. Furthermore, clinical trials conducted in health services often require assistance from departments such as finance, pathology and medical imaging. However, there were reports of delays, errors or omissions in processing trial-related requests due to a lack of in-depth understanding of clinical trials. The inconsistent knowledge and experience about clinical trial conduct has contributed to inefficiencies across a health service.

Organizational research mentality and culture within a health service were strongly reported to align with research outputs. A renowned oncology research institute in Melbourne was mentioned repeatedly as having an ideal clinical trial culture, where clinical trials were embedded into their standard care and vision. Despite this, research culture, or lack thereof, was the most consistently reported challenge faced by sponsors, researchers and clinicians wanting to conduct clinical trials within a health service. This was described in terms of trial activity driven by people, not systems; overcomplicated and unnecessary layers of research governance and risk minimization; delays obtaining departmental approvals; and lack of tangible support such as research space, recognized research workforce, funded permanent research positions, dedicated clinician research time and departmental cooperation. All participants desired more engagement and commitment on the part of health service boards and executives to create a supportive environment. This would involve a well-considered research strategy involving every department and levels from the executive directors through to front-line staff and be accompanied by investment. To be confident in this investment, one passionate clinician researcher urged that investors need to engage and understand the return of the investment made:

The first is, return on investment is huge. And the second is, it will not come back to the place it was spent […] and we shouldn’t expect it to. […] The third thing is that, if we pay attention to and resource the potential returns and investment properly […] the whole health system will not only get those benefits but know where they went. (Clinician Researcher 6)

### Clinical trial capacity and capability is highly variable across RRR regions

Participants reported significant variability in health service clinical trial activity, capacity and health service maturity across RRR health services in Victoria, Queensland, South Australia, Western Australia, Tasmania and the Northern Territory.

Victoria was reported to have purpose-built oncology research centres and centralized cross-disciplinary clinical trial units in several large regional towns, grown and supported through a range of capacity-building initiatives for many years with great success. However, the clinical trial capability in smaller rural Victorian hospitals was reported to be limited due to inconsistent adoption of the Ethical Review Management (ERM) online platform and lack of permanent clinicians. Queensland was reported to have clinical trial activity in few large regional tertiary health services, although one participant reported:

You can’t even look at a (Queensland) health service and think: “oh, they do trials.” It is all pockets. […] It (clinical trial activity) may not necessarily be coordinated across a whole health service. (Health Service 11)

In other states, clinical trial activity outside the capital city was reported to be very low or zero. While some health service and industry participants felt there had not been enough awareness and interest from clinicians to bring trials into RRR Australia, the clinician researchers interviewed reported it was not for lack of desire but instead lack of capacity or support:

I don’t take them because we can’t – even though it’s good for patients, but we can’t because we have no resources to support [us running clinical trials]. (Clinician Researcher 5)

Some regional clinicians also report frustration over not being able to provide trials to their patients:

There are good (RRR) clinicians doing work in this space on their own, not supported […] for lots of years. And they’ve tried to build their case and tried to ask for what they knew they needed but have not had it. […] outside of cancer, it hasn’t really happened. (Health Service 11)

Despite these frustrations, there was a reported sense of optimism about the future of clinical trials in RRR Australia, with national reforms streamlining human research ethics reviews, national governance frameworks and wider adoption of the ATM making it easier to establish clinical trials. Furthermore, participants from clinical trial networks and collaboratives reported they had begun including equitable access as a key priority area in their strategic plans with the aim to increase regional and rural sites in their trials.

### Clinical trial participant recruitment

#### Always challenging but work being done to break down the barriers

Participants reported four key enablers to reaching clinical trial participant recruitment targets: source of eligible and engaged patients; effective mechanisms in place to identify, screen and enrol participants; experienced organized and engaged team; and an enabling study design with flexibility to adapt. Successful recruitment mechanisms reported included clinical trial networks, patient registries and fit-for-purpose medical records. In addition to these mechanisms, having an experienced, engaged and organized study team was important and created an efficient and smooth recruitment experience for both trialist and participants. Factors reported to negatively impact participant recruitment and retention included participant burden, unnecessarily strict criteria, lack of awareness and scepticism of clinical trials on the part of patients and lack of cultural and language considerations. Participants working in management roles within research institutes felt that many recruitment barriers could be overcome with prior consumer engagement, proper protocol planning and the ability to monitor and identify early challenges and adapt the study accordingly.

#### Unique challenges in RRR health services but participant retention might be better than metropolitan sites

Participants reported additional participant recruitment challenges in RRR health services such as practical implications for participants to travel vast distances, perceived low awareness of clinical trials on the part of patients and clinicians and the need for clinical trials to be meaningful to the community.

Currently, the distance required for patients to travel from a remote health service to take part in a trial was reported as a significant barrier to trial participation. One clinician researcher described the onerous journey for one participant:

You need a day to get to [regional location] airport […] stay in [regional location] for a night. And then you’ve got to take a plane, three or four hours down to [state capital city] and find accommodation. So, you’re really losing almost three full days away from home […] And then fly all the way back. (Clinician Researcher 3)

In addition to the burden of distance, sending patients away to another location to take part in a trial was reported as impractical and disruptive to their care. Two regional clinician researchers in oncology reported that, although offering clinical trials was considered best practice in oncology care, it was not practical to send all patients to cities:

[…] So that means, 95% of my patients, I need to send them away [to cities] for trials [...] So that’s what the implication is. (Clinician Researcher 2)

It’s quite frustrating for us and for the patients that they have to go somewhere else. The thing that motivates me to, despite all the clinical work, is to access more trials here so that we can offer more treatment locally for the patient, you know, close to their home without travelling […] 1,000 km from where we live. (Clinician Researcher 4)

Some participants had a perception that clinicians in RRR health services did not talk to their patients about being involved in clinical trials, and therefore there was lack of awareness of clinical trials. However, participants from RRR health services reported that clinical trials should be promoted through the community or health service on the basis of community priorities rather than relying on the interests or capacity of busy individual clinicians. Some participants believed recruitment might be better in RRR communities that have a “village mentality” (Health Service 1), where the community is willing to support and promote trials meaningful to them. One metropolitan industry participant reported to have had a positive recruitment experience in regional centres, with retention of trial participants being “on par or better than recruitment in cities.” (Industry 4).

It was reported that First Nations’ cultural factors were often overlooked in industry-sponsored trial protocols, and needed to be considered to build trust and safety within Aboriginal and Torres Strait Islander communities.

### Clinical trial approval processes

#### The approval process is long and variable across Australia

Most participants reported the process of obtaining Human Research Ethics Committee (HREC) approval and Research Governance Office (RGO) authorization for clinical trials conducted in a public health service was long, with inconsistency both between and within states and territories and individual health services.

Participants reported that Australia is currently in the midst of a national clinical trial reform agenda evidence by reports of a range of clinical trial capability building initiatives. Specific initiatives mentioned included “encouraging more clinical trials in Australia” (Health Service 12), where every jurisdiction received federal funding to increase clinical trial capability and facilitate coordinated clinical trial support tailored to their needs; the “National Mutual Acceptance scheme [for ethical and scientific review of multi-centre research]” (Health Service 2), to reduce duplication of ethical review of multi-centred research; the “National Clinical Trials Governance Framework” (Health Service 14), which articulates standards and responsibilities to ensure quality and safety of clinical trials within health services; and the National “One-Stop-Shop” (Health Service 14) to develop a national site-specific assessment (SSA) to streamline research governance approval processes. Participants reported that the HREC approval process has improved in recent years due to the national mutual acceptance accreditation scheme, which enables accredited HRECs to review multi-centre research, but there were still instances of duplication by private or higher education institute HRECs that are not accredited. Additionally, research involving First Nations Australians requires additional approvals that vary across jurisdictions.

Most participants reported issues at some point throughout the RGO application process. While the general components of the SSA were similar across jurisdictions, significant variations in the preparation, review and execution of these components were reported. Firstly, every jurisdiction had a different online platform for SSA applications with varying steps, information and application fees. For example, despite Queensland and Victoria both using Ethical Review Manager, an online platform to manage ethics and governance authorizations, it was reported that not all health services across Victoria consistently used Ethical Review Manager. Secondly, administrative variations between health services within the same jurisdiction were reportedly frustrating for researchers who needed to obtain authorizations for multi-centred trials. Thirdly, at a health service level, the knowledge, capacity and experience of the RGO staff, and the organizational research culture were reported to impact the approval process experience. For example, obtaining sign off from head of departments and business managers was frequently reported to delay SSA submissions by weeks. In addition, negotiating the content of the clinical trial research agreement (that is, clauses, payments and special conditions) between health service and sponsor was reported to be the most time consuming. It was not unusual for contractual negotiations to take weeks to months with some states and territories and sponsors requiring amendments to clauses and schedules in the standard Medicine Australia clinical trial research agreement. The clinician principal investigator’s knowledge and understanding about the RGO process, contracts and budgets was also reported to impact negotiations. For example, there were reports of clinicians agreeing to conduct trials with little to no funding, making it unfeasible for the health service to support the trial. Several health service participants reported they now have in place trial managers, investigator training and a standard schedule of fees for clinical trial services to support investigators and streamline processes.

Finally, there were conflicting opinions between health service and clinician researcher participants. Many clinician researchers felt RGO process were “varied levels of awful” (Clinician Researcher 6), “spiraling out of control” (Industry 4), “policing” (Clinician Researcher 1), “low value” (Clinician Researcher 2), “driven by risk management” (Industry 4) or the “most regulated thing there is. Period.” (Industry 2), or that it “wears researchers down” (Industry 5). However, health service participants felt some clinicians lacked knowledge of the guidelines, institutional processes and legal requirements needed to conduct a clinical trial, leading to delays and conflict with the RGO. In addition, health service participants acknowledged the total time from submission to approval may blow out more than anticipated by applicants; however, most of the time the RGO review period still fell within the RGO target turn-around approval times (“clock time” (Health Service 10); the time the application sits with the RGO through to action). Furthermore, according to health service participants, the RGO approval process became easier with experience, and they have always been supportive of researchers and research.

#### Lack of clinical trial maturity is an additional challenge for RRR health services

Regional health services with university affiliation and frequent clinical trial activity reported to have more experienced RGO staff than rural and remote services but smaller RGO teams than metropolitan health services. In rural and remote health services, the size and capacity of the RGO teams was reported to range from limited to non-existent, with inadequate resources to properly assess SSAs for clinical trials. Again, workforce management was a consideration: “If we’re going to […] work in that space, how do we provide support and cover when that one RGO [officer] with no support team has to take leave?” (Health Service 11). Acknowledging the limited RGO capacity in rural and remote health services, there was reported hope that the ATM would reduce the workload by using the “primary sites to do the legwork for those kinds of approvals.” (Clinician Researcher 3).

## Discussion

This study is the first to explore the perceptions and experiences of clinical trial stakeholders about the Australian clinical trial landscape with a focus on RRR health services. This discussion highlights the barriers and enablers identified in the findings that should be considered by the ATP and any other initiatives aimed to develop clinical trial capacity and capability in RRR health services at local, state and national levels. In general, it was reported that health services located in metropolitan regions were better supported with more skilled and qualified staff, equipment and services and participants in proximity. The general challenges, which were particularly relevant to the broader Australian clinical trial landscape and affected both metropolitan and RRR health services clinical trial capability, involved unstable clinical trial funding and investment, lack of recognition for the trial workforce and long and inconsistent approval processes. RRR health services faced additional issues in conducting clinical trials (lack of space, infrastructure and skilled trial staff, competition with clinical services and time-sensitive or temperature controlled logistics) while needing to also overcome the inherent challenges of healthcare delivery to small populations over vast geographical areas while experiencing chronic workforce shortages [21]. Despite these challenges, participant recruitment and retention in RRR areas was reported to be on par or better than metropolitan sites, with RRR clinicians reporting a high appetite to be involved and offer clinical trials to their patients.

**Fig. 1:**
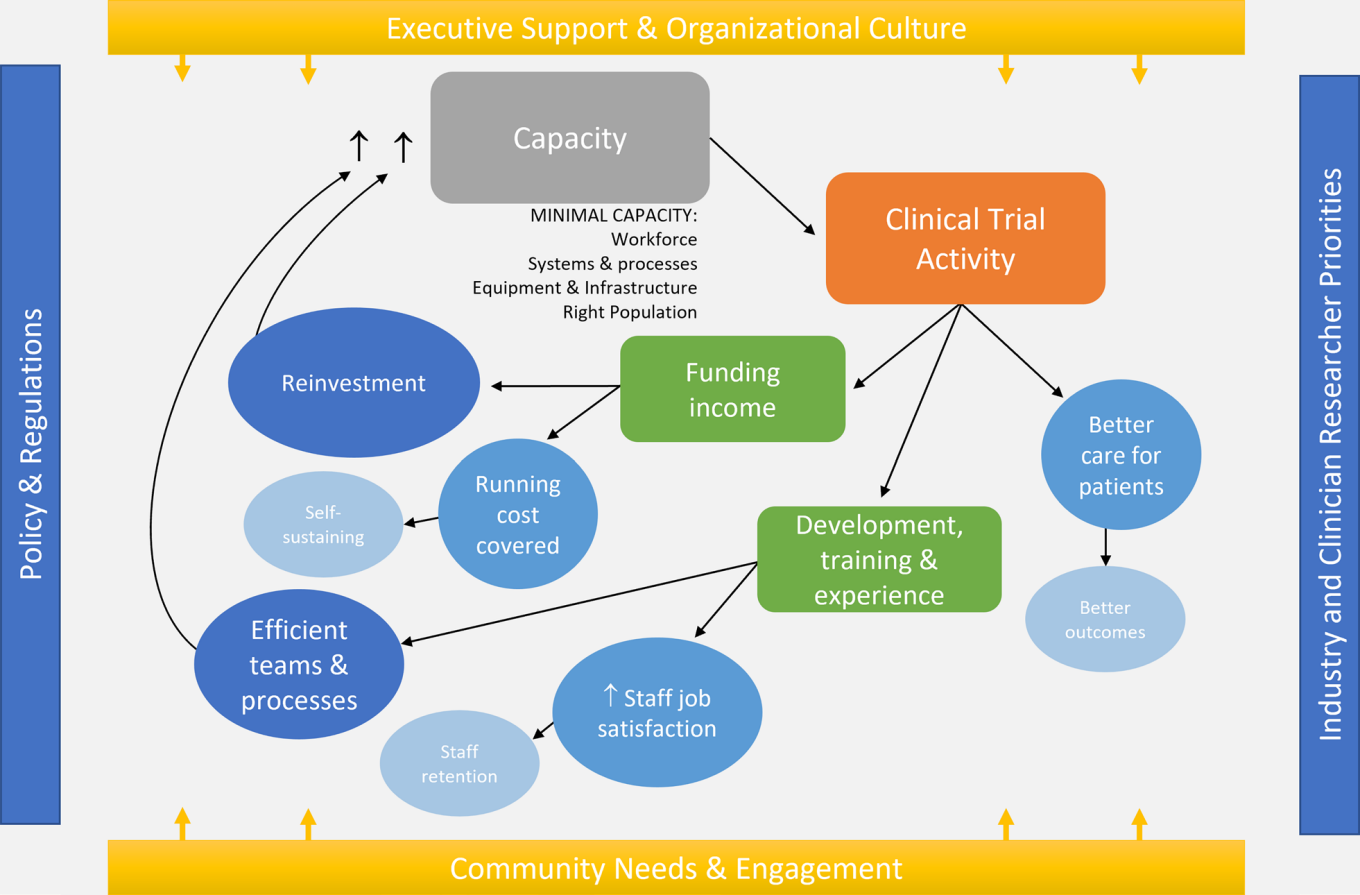
Clinical trial capacity within a health service and the impacting contextual factors

This study provided insights into the inner and outer contextual factors influencing the capacity of RRR health services to conduct clinical trials as conceptualized in Fig. 1 and further discussed in the remainder of this discussion..

Ultimately, from the findings, a RRR health service required a minimum level of capacity to conduct clinical trials that are safe for patients and meet regulatory standards. This included: (i) suitable and available workforce for upskilling; (ii) functioning systems and processes to support clinical trials management, operations and governance; and (iii) access to relevant equipment, services and infrastructure and the right population pool(s). To facilitate a supportive and stable environment for clinical trial activity, strategic executive leadership and investment and organizational culture was essential. Growing capacity was reported to be driven by industry and clinician researcher priorities, where the most meaningful clinical trial activity considers community priorities as understood through engagement and consultation with all relevant stakeholders.

### Clinical trials benefit patients

This study observed that clinical trial patients are perceived to receive better care and can have better health outcomes as a direct result of being closely managed, monitored and receiving uniform treatment in a clinical trial, which is consistent with other studies in the field [2, 22]. In addition, several Australian studies attest to the willingness of RRR potential participants to take part in trials. For example, a study by Sabesan et al. [6] showed that rural and remote oncology patients would be interested in participating in a clinical trial if they were offered one; however out-of-pocket patient costs and need for a support network were the main barriers to taking part if they had to travel long distances. Two Australian studies have demonstrated that RRR participants taking part in oncology clinical trials at their local health service (regardless of via telehealth or the standard model) reported high levels of acceptability in terms of quality of care and reduced burden (cost, time, travel and support) associated with travelling to metropolitan study centres [7, 8]. This acceptability of clinical trials by people living in RRR Australia is encouraging and demonstrates significant potential for successful trial delivery in these areas. These studies focused on oncology trials and more research is needed on the perceptions of non-oncology trial participation.

This study also highlighted both challenges and opportunities of involving First Nations people in clinical trials. It was reported that in many RRR health service settings, First Nations people are over-represented in clinical presentations, but significantly under-represented in clinical trials. Participants reported that more needs to be done to ensure clinical trial protocols and resources are culturally appropriate. This finding is also supported by a study by Cunningham and Garvey [23], who found potential systemic barriers to First Nations people’ oncology clinical trial participation based on the study design, study location, cancer type and inclusion criteria of registered clinical trials in Australia. The author encouraged purposeful review of study protocols that by design could potentially marginalize and exclude First Nations people. It was recommended by participants of this study that community consultation, while it might not be a standard activity in most clinical trials’ startup phase, is essential in respectfully engaging, involving and recruiting First Nations people in clinical trials. Furthermore, First Nations people desire to receive care closer to home but experience anxiety, loss of agency and spiritual and social costs in accessing cancer treatments, adding to the importance of removing barriers to clinical trials for First Nations people [24]. It is important that clinical trial activity is driven by the needs of the community, especially in tight-knit RRR communities, to ensure efforts are not wasted on trials that offer no benefit or are not considered to have sufficient priority.

### Clinical trial activity drives growth in capacity and capability

The volume of clinical trial activity was reported to drive both capacity and capability-building through staff training, system development and income generation. Clinical trials brought revenue into the health service, with some generating profits from commercially sponsored trials. When clinical trials were well managed and a steady incoming pipeline of trials was fostered, health services were reportedly able to reinvest the profits into building capability and capacity, including growing teams, extending staff contracts and purchasing equipment. Therefore, the development of clinical trial business knowledge and skills within health services is important for sustainability. However, to develop this level of sustainability requires initial investment to onboard staff, develop processes and cover any loss incurred during the initiation phase of establishing clinical trial capacity.

In addition to providing a source of income, clinical trial activity was a source of training, professional development and experience, mostly driven by sponsor-led protocol-specific training and mandatory GCP training, ensuring that trials are conducted according to the protocol and regulatory standards. This training and experience reportedly increased job satisfaction of both clinical trial professionals and clinician researchers. This finding is consistent with the work of Francis et al. [25], who found that 92% of Australian medical oncology trainees considered access to clinical trials an incentive to attract oncology registrars to rural work. In addition, the 2021 Australian and New Zealand College of Anaesthetists regional and rural workforce strategy recognizes clinical trial involvement is a “rewarding and vital part” of an anaesthetist role and acknowledged the need for trials to be supported in RRR hospitals [26]. Therefore, it might seem that trial activity may assist in attracting and retaining specialist doctors in RRR health services – an important workforce strategy that may support clinical trial capability.

In addition to job satisfaction, repetitive trial activity was reported to drive trial-specific efficiencies (for example, faster study startup phase, faster recruitment rates and retention and better data quality) as a result of continuous learning, training and process refinement. The learning curve theory would suggest that, with each trial, the efficiencies of individuals, teams and organizations increase [27]. The greatest rate of learning and capacity-building occurs at the start and slows as experience and efficiency develops [27]. Factors such as staff turnover (losing experience), sharing of knowledge, incentives, cumulative trial activity and institutional culture can enhance, disrupt or impede this process [27]. Considering the significant current challenges facing RRR health services, the rate of capacity-building and learning is likely to vary and be dependent on baseline clinical trial maturity, workforce stability, initial trial activity and support from the executive leadership. The ability of a health service to facilitate and cultivate a culture that supports research activity is thus critical to the development and sustainability of clinical trial capability and capacity. This is not a unique viewpoint, with Practice Standard 1 of the 2022 Royal Australasian College of Medical Administrators position statement on remote, rural and regional medical leadership by medical administrators [28] stating:

Remote, rural and regional health services should not just be “receivers” of research and outreach services. They should be leaders of innovative research given the different nature of the context of care provided, and the unique nature of the workforce and challenges found. (Practice Standard 1)

### Executive leadership is critical to building and sustaining capacity and capability

Lack of executive leadership, governance and organizational research culture were significant perceived barriers to efficient, stable, consistent and sustainable clinical trial capacity and capability within health services. The toll of these challenges discourages clinicians from becoming involved in clinical trials. Organizational culture as a geographically dependent driver of research activity has been previously described, and requires a holistic approach to improve RRR research culture at individual, team, organization and state levels [29]. At the organization level, executive teams can engage with their staff and community to develop a research strategy that prioritizes clinical trials on basis of the needs, ability and capacity of their health service. However, a strategy on its own is insufficient to make change. A study by Edelman and others [30] on the research impact of a regional Queensland health service found that, while executive support, research strategy and support existed, there were missing links across the health service that still required individuals to facilitate and champion research. Therefore, for a strategy to be effective, the executive committee needs to drive this strategy through all the levels of the health service so all staff and departments (for example, clinical governance, business and finance, allied health, operation support, medical, surgical, etc.) realize and engage with clinical trial activity as opposed to champions needing to drive them independently.

### Bringing trials closer to home will require a whole system and collaborative approach

Increasing access to clinical trials for people living in RRR regions will likely require not only addressing resource and workforce limitations at RRR health service level but also addressing general issues affecting the clinical trial landscape. This includes harmonizing the clinical trial authorization process, recognizing the clinical trial workforce with career pathways, and embedding efficient, sustainable, valued clinical research in all levels of the health service activity. These complex issues require investment, collaboration and reform at national, jurisdictional and individual health service levels. Initiatives in this space, as mentioned in this study, are evolving rapidly, necessitating attention to ensure all changes are collaborative, beneficial, expansive and avoid duplication. Ultimately, however, sustainable reform to bring trials closer to home requires an approach that considers evidence-based implementation strategies across all levels of the health service and government structure.

### Strengths and limitations

The influence of the different sampling techniques on the findings could not be determined; however, each participant was well positioned (median of 14 years’ experience in the clinical trial sector) to provide in-depth responses to the interview questions, with representation across all jurisdictions and stakeholder groups. While the main themes were largely consistent across each jurisdiction, due to the large volume of data collected and heterogeneous level of representation from each jurisdiction, it was not possible to detail or define the landscape according to each state or territory within this manuscript. In addition, further exploration of the clinical trial reform agenda, particularly around outcomes and impact, could be warranted, as there were several initiatives reported to have occurred over the last decade; however, not all of these were not consistently reported.

## Conclusions

The clinical trial landscape across Australian health services is heterogeneous in terms of the quality and availability of clinical trial training, stable workforce capacity, executive support and research culture, site capability and clinical trial approval processes. RRR health services report low to no clinical trial activity or capacity, with regional cancer centres being an exception. The ATP, through carefully positioned jurisdictional RCCCs, has an opportunity to support the uptake of the ATM, through clinical trial capacity and capability-building pillar activities that are suited to the needs and contextual factors within each jurisdiction. This program has potential to address many of the barriers found in this study while enabling patients to safely access trials closer to home, and it is recommended that these are considered during ATP implementation planning and execution. A key part of the ATP is embedding clinical trials into health services. To achieve this will require commitment and engagement by executive leadership to stabilize capacity and cultivate a research culture. As the ATP is one program among several initiatives currently underway to address key challenges affecting the clinical trial landscape, it will be critical that a whole of system and collaborative approach is taken to avoid inefficiencies and segregation.

## Supplementary Information

Additional file 1.

Additional file 2.

## Data Availability

The interview guides used in this study have been provided as an Additional file 1. Data have been obtained for consultancy and are not available at this stage.

## Abbreviations

ATM: Australasian Teletrial Model
ATP: Australian Teletrial Program
GCP: Good Clinical Practice
HREC: Human Research Ethics Committee
MMM: Modified Monash Model
RCCC: Regional Clinical Trial Coordinator Centre
RRR: Rural, Regional and Remote
RGO: Research Governance Office
SSA: Site-Specific Assessment
