# Supplementary material for: The Australian clinical trial landscape: Perceptions of rural, regional and remote health service capacity and capability
Source: Health Res Policy Syst. 2024 Dec 19;22:171. doi: 10.1186/s12961-024-01270-z (PMC11657177; doi:10.1186/s12961-024-01270-z)
Supplement: Supplementary file 1 — Additional file 1. [file 12961_2024_1270_MOESM1_ESM.pdf]

The Australian clinical trial landscape: Perceptions of rural, regional, and remote health service capacity and capability

Title: **Additional File 1** – Interview Guides

Description: This file contains the interview guides that were used to conduct the semi-structured interviews. The first guide was used to interview clinician researchers and industry stakeholders. The second guide was used to interview health service stakeholders.

**Interview Guide – Clinician Researcher and Industry Stakeholders**	
**Landscape & Background**	1. **What is your current role in clinical trials?**<br>2. **Can you tell me about the clinical trials you have been involved in up to this point?**<br>Prompts:<br>o What type of clinical trials are they?<br>o Are/have they been multi-centred?<br>o Where are/have your sites been located?<br>3. **Do you have any knowledge about the Australasian Teletrial Model?**
**Education & Training**	4. **Do you feel researchers, clinicians and research staff generally are adequately trained and experienced to carry out clinical trials?**<br>Prompts:<br>o If not, where/why do you feel the knowledge or training is lacking?<br>5. **In your opinion, what training should clinical trial researchers, clinicians and research staff have?**<br>Prompts:<br>o How can they access this training?<br>o What would help staff access this training?<br>o What clinical trial training does your institutions/workplace provide?
**Workforce**	6. **What is your opinion on the current workforce in clinical trials in terms of demand, employment conditions and career opportunities?**<br>7. **What is your opinion on how to attract, support and retain clinical trial workforce in the clinical trial sector?**<br>Prompts:<br>o What are the challenges to doing this? attracting/supporting/retaining<br>o How do the challenges you discussed apply in the RRR setting do you think?
**Equipment**	8. **Do you require specialised equipment to conduct your clinical trials? If so, what are they and how is this equipment usually arranged and funded?**<br>Prompts:<br>o Do you usually arrange the use as in-kind, a part of standard care or are they costed?<br>o How would this impact your decision to run clinical trials in RRR regions?
**Site Recruitment**	9. **In your opinion, what makes a clinical trial site successful?**<br>10. **What is involved in selecting and setting up a clinical trial site?**<br>Prompts:<br>o Who decides what sites will be selected and how it this decision made?<br>o What are the main challenges to adding/setting up sites and retaining them? And what would make this process easier?<br>o How would these challenges be different for sites in RRR regions?
**Participant Recruitment**	11. **In your opinion, what drives participant recruitment?**<br>Prompts:<br>o What are the challenges to enrolling participants?<br>o What helps participants stay in a clinical trial, and not drop out?<br>o How is taking part in a clinical trial different for someone living in a RRR area?
**Policy & Process**	12. **What approvals do you require before starting one of your clinical trials?**<br>Prompts:<br>o Have you found obtaining any of these approvals challenging? If so, please explain the challenge.<br>o What could make this process easier for you?<br>13. **Can you tell me what reporting you are required to do while carrying out a clinical trial?**<br>Prompts:<br>o Have you found any reporting requirements or reporting process challenging?<br>o What could make this process easier for you?
**Teletrial Model**	14. **How do you feel about clinical trials being run in a decentralized way using the Australiasian Teletrial Model?**<br>Prompts:<br>o How will this change the clinical trial landscape?<br>o What do you think will be the challenges to adopting this model?<br>o What new opportunities will this model bring do you feel?

**Closing**	**That is the end of the consultation questions. Do you have anything else to add that we did not discuss earlier?**

**Interview Guide – Health Service Stakeholders**	
**Landscape & Background**	**1. Can you tell me what your role/involvement is currently in clinical trials?**<br>**2. Can you tell me about what your experience has been like in clinical trials up to this point?**<br>**3. Do you have any knowledge about the Australiasian Teletrial Model?**
**Education & Training**	**4. Do you feel researchers, clinicians and research staff generally are adequately trained and experienced to carry out clinical trials?**<br>Prompts:<br>o If not, where/why do you feel the knowledge or training is lacking?<br>**5. Does your institution provide or support clinical trial training to staff?**<br>Prompts:<br>o If so, what type of training is provided and how?<br>o If not, what type of training would be useful?
**Workforce**	**6. What is your opinion on the current workforce in clinical trials in terms of demand, employment conditions and career opportunities?**<br>**7. What is your opinion on how to attract, support and retain clinical trial workforce in the clinical trial sector?**<br>Prompts:<br>o What are the challenges to doing this? attracting/supporting/retaining<br>o How do the challenges you discussed apply in the RRR setting do you think?
**Equipment**	8. **What are your observations on how well clinical trials use existing equipment and infrastructure within the health departments/hospitals.**<br>Prompts:<br>o Is use of equipment and resources for clinical trials generally welcomed and shared? And who decides? Please explain drawing from experience if possible.<br>o What factors impact whether or not equipment can be used in a clinical trial?
**Site Recruitment**	**9. What are the factors are important for sites to consider when approached to take part in a clinical trial?**<br>Prompts:<br>o During site assessment and selection process, what factors determine the speed at which this is done? For example, what factors speed up and slow down this process?<br>o What are the main issues, repeated issues, or sticking points to site selection and set up?<br>o What would make this process easier? For example, what support or changes are needed?
**Participant Recruitment**	**10. Can you tell me how your institution performs in relation to recruiting trial participants? Do your studies consistently meet recruitment targets?**<br>Prompts:<br>o What factors (positive and negative) impact participant recruitment?<br>o What factors impact a participant staying in a clinical trial until the end?
**Policy & Process**	**11. Can you tell me what your experience has been like navigating the clinical trial approval, contractual and reporting process with industry or investigator lead researchers?**<br>Prompts:<br>o What is the average approval time from submission to approval?<br>o What are the main reasons for longer approval times?<br>o What could help shorten the approval time?<br>o What are common, repeated or sticking points along the regulatory pathway that you find many applicants get held up?<br>o What can be done to make this easier?
**Teletrial Model**	**12. How do you feel about clinical trials being run in a decentralized way using the Australiasian Teletrial Model?**<br>Prompts:<br>o How will this change the clinical trial landscape?<br>o What do you think will be the challenges to adopting this model?<br>o What new opportunities will this model bring do you feel?
**Closing**	**That is the end of the consultation questions. Do you have anything else to add that we did not discuss earlier?**
