# Supplementary material for: The Australian clinical trial landscape: Perceptions of rural, regional and remote health service capacity and capability
Source: Health Res Policy Syst. 2024 Dec 19;22:171. doi: 10.1186/s12961-024-01270-z (PMC11657177; doi:10.1186/s12961-024-01270-z)
Supplement: Supplementary file 2 — Additional file 2. [file 12961_2024_1270_MOESM2_ESM.pdf]

The Australian clinical trial landscape: Perceptions of rural, regional, and remote health service capacity and capability
Title: **Additional File 2 –** Supplementary Table of major themes and supporting quotes of the clinical trial landscape in Australia generally and specific to RRR areas.

**Supplementary Table: Major themes and supporting quotes of the clinical trial landscape**

| Interview topic | Themes<br>▪ Sub-themes | Example quotes |
|---|---|---|
| Education and training | **Variation in clinical trial training received with Good Clinical Practice (GCP) the minimum standard.** | |
| | ▪ On the job training was reported to be the most common form of training. | *"You know, a lot of it is just experienced based […] I don't think you can really understand the entire workings of the clinical trial until you've worked in it and gone through it." (Industry 5)* |
| | ▪ Learning environment for training depended on types of trials and the people in the environment. | *"A few people that were thrown in the deep end to pick up clinical trials that probably weren't sufficiently trained and then have had to learn to swim pretty quickly." (Health Service 9)*<br><br>*"If you don't have those commercial quality drivers to drive quality into your process, and you don't have the experience of the larger units that potentially can assist by providing that training, then you tend to see a slightly lower level of quality." (Health Service 16)* |
| | ▪ GCP training and study protocol training were reported to be the minimum standard training required for site trial staff. | |
| | **Clinical trial training is relative to clinical trial activity, and thus limited in RRR.** | |
| | ▪ RRR health services without active trials did not have exposure to or need for staff training. | Quote in text |
| | ▪ More sharing of knowledge, mentoring and training is needed in RRR health services where clinical trial activity is developing. | |
| Workforce | **Trial workforce challenges are complex, persistent and across the board.** | |
| | ▪ Competitive funding models contributes to insecure and fractional work for trial staff. | *"They had clinical trials and the money ran out. […] The money ran out to fund the nurse but the project continued, eventually they had to close up the project at the site." (Health Service 9)* |
| | ▪ Lack of professional recognition and career progression affects the attraction and retention of clinical trial staff. | *"So [the] research nurse is sometimes doing the coordinator role at the N2 level or some other units having a research nurse at the N5 level. […] there is no standardized career pathway kind of thing." (Health Service 2)* |
| | ▪ Clinician researchers feel under supported due to increasing clinical workloads and, lack of protected and remunerated research time. | *"[…] It's just so much red tape, bureaucracy, admin time, a lot of it is all in kind. You know, they're (clinician researchers) doing this on top of their professional paid jobs […] that's incredibly frustrating." (Industry 5)* |
| | ▪ More support is needed to stabilize the clinical trial workforce. | *"[…] If research is prioritized within every hospital, and therefore positions are made available as part of the normal infrastructure then these (workforce) issues could be mitigated." (Industry 13)* |
| | **Unique workforce challenges in RRR health services exist alongside an appetite for trials.** | |
| | ▪ Low to no clinical trial workforce in RRR health services with variation across departments. | *"Cardiology we've only got one research nurse at the moment, […] So cardiology is very low. But I think in cancer we have about 4 to 3 full time equivalent people, plus the admin support." (Clinician Researcher 5)* |
| | ▪ Severe workforce shortages risks destabilizing clinical trial capacity in RRR health services. | Quote in text |

| | | |
|---|---|---|
| | ▪ Call for trial staff to be consolidated across whole of RRR health services. | |
| | ▪ RRR clinicians reported being passionate about providing their patients access to a clinical trial. | *"We all feel passionate that the demographic and the patients that we serve are actually being disadvantaged by not having access to clinical trials, but also by not being represented in clinical trials." (Clinician Researcher 8)* |
| **Equipment and services** | **Metropolitan sites generally have access to more resources to conduct clinical trials.** | |
| | ▪ Well established health services that have partnership with research institutes are better resources to run clinical trials. | *"So, they have like a PET MRI, another MRI, I think a CT scanner, we have like within our clinical trial group, we have EKG machines, we have an EEG machine downstairs […] So the imaging centre is a […] three-way partnership between [three institutes]." (Industry 5)* |
| | ▪ Unpartnered health services or siloed research groups are likely to face challenges of sharing equipment and services with routine clinical care. | *"Access to clinic rooms and equipment for just the basic [clinical trial] assessments was really hard to come by." (Industry 13)*<br><br>*"[…] Usually [the hospital] don't have a [medical imaging] booking for the next six months. But you know [in a] clinical trial, you need these every 12 weeks." (Health Service 7)* |
| | ▪ Unexplored issues with clinical trial digital infrastructure. | *"Digital infrastructure is essential. I think that's a real glaring omission. That it is not just a computer, but the software to be able to run clinical trials…having external sponsors being able to remotely […] monitor all that data is […] a critical function [...] The discussion around digital infrastructure is so minimal." (Health Service 16)* |
| | **By contrast, RRR health services are insufficiently resourced for clinical trials.** | |
| | ▪ Lack of physical space, specialised services, and capacity of services limited clinical trial capacity in RRR health services. The degree of limitation increased with remoteness. | *"They don't have ultra-low temperature capabilities, or they might not have, you know, a swing bucket centrifuge for blood processing." (Health Service 15)* |
| | ▪ Regional university infrastructure might provide opportunity for providing space. | *"There is no infrastructure for research apart from […] the buildings that the universities have put in place." (Health Service 13)* |
| **Clinical trial sites** | **An enabling culture, capacity and system is essential to running a successful site.** | |
| | ▪ Well established clinical trial units are currently funded by a combination of grants and commercial sponsored funding. | *"[…] Investigator initiated trials costs (more than what is funded), [it] never pays for everything, not even client. Industry trials actually allows the sites to cover their costs and walk [with] a little bit away" (Industry 4)* |
| | ▪ Understanding clinical trial business is important for all health services wanting a sustainable and financially stable trial site. | *"They (sponsor) offered him (clinician) $30,000 a patient, he said, I'll do it. I'll just do it. And we've got nothing in the budget. So there's absolutely nothin, 200 bucks or something […] operationally they can't run it." (Health Service 4)*<br><br>*"They actually took financial responsibility to receive money and they created fabulous processes that enabled the trial unit for billing to have the correct billing to happen." (Health Service 5)* |
| | ▪ Research culture of the health service is essential to a productive and sustainable clinical trial site. | *"You know, [metropolitan oncology center], they have an amazing culture for research. Every single patient that has cancer is put on a clinical trial, and that's just their mandate… Whereas other institutions would consider a clinical trial if the patient had no other options …that difference in mentality leads to recruitment challenges." (Industry 13).* |
| | ▪ Investment, engagement, and support by executives/directors is needed that aligns with a well-considered research strategy. | *"He (the CEO of a health service) was surprised when I told him clinical trials improve health outcomes …clinical trials reduce variation in practice… minimizes wastage of investigation… improves collaboration* |

| | | |
|---|---|---|
| | | *between patient and the families and local system.… improves [patient] health literacy, …professionalizes the workforce… it brings money… he was surprised to hear all of it." (Clinician Researcher 5)*<br><br>*"I'm talking about conscious decision about allocating 2 or 5% or whatever it is the organization's operating budget for research, having that aligned to a strategy so you can demonstrate that the research that you were doing brings back the value to the organization as well as to potentially the broader communities" (Industry 14)* |
| | **Highly variable clinical trial capacity and capability across RRR regions.** | |
| | ▪ Low to no levels of clinical trial activity outside capital cities, particularly in NT, WA, SA, TAS. | *"So, at the moment, I think we got 44 full clinical trials active across NT (Northern Territory) Health and then 43 of those [are in the capital city] if you drop Alice Springs Hospital." (Health Service 3)*<br><br>*"We do very little clinical trials in [Western Australia's] regional areas because we don't have the infrastructure." (Clinician Researcher 3)*<br><br>*"There are no big clinical trials occurring at the moment in South Australia in the regional areas." (Health Service 12)*<br><br>*"The north [of Tasmania] don't actually have that much research activity. 80 per cent of the research activity is in the south (where the capital city is)." (Health Service 9)* |
| | ▪ Frustration felt by RRR clinicians who are not able to provide trials to their patients due to lack of site capacity. | *"They [clinicians] realize they are missing out […] they can't enable their patients to access clinical trials without them essentially being handed over to another doctor in the city. […] So, there's a pent-up latent appetite for clinical trials." (Health Service 13)* |
| | ▪ Growing optimism and momentum for more equitable access to clinical trials at RRR sites. | *"[…] Once we get the results back from the feasibility survey, we would review it with our research operations committee and try to establish a split (in trial sites) between metropolitan and regional, rural, remote areas, so that there's equitable access and opportunity […]" (Industry 13)* |
| **Participant recruitment** | **Always challenging but work is being done to break down the barriers.** | |
| | ▪ Streamlined and efficient processes to identify, recruit and involve participants is important to successful recruitment. | *"One of the key things is, you know, identifying sites that have those patient registries, you know, lists of patients who are happy to be contacted to be involved time and time again." (Health Service 10)*<br><br>*"We recruit 80 patients in [a] Saturday morning, we would have, station one […], informed consent, next station […] analysis and OBS, next station […] their vaccination. Then they'd […] wait 30 minutes after the vaccination. […] we supply newspapers […] snacky foods and stuff like that […] And generally, they'd have a good time […]. So having a really good eye for, you know, organizational ability." (Clinician Researcher 1)* |
| | ▪ General challenges to recruitment include participant burden, strict criteria, attitudes towards trials and language and cultural barriers. | *"I have experience [of participants] pulling out of trials because […] the burden on the participant is just too great. […] Especially if we're looking at certain age ranges or disease processes, sometimes these people have a lot of other things medically going on" (Industry 5)*<br><br>*"We don't traditionally involve our non-English speaking patients in our clinical trials enough because […], you've got to have […] an accredited translator, and all the documents gotta be translated backward and forward" (Industry 6)* |
| | ▪ Thorough consumer engagement, careful protocol planning, monitoring risks and adapting when needed, | *"If they're if they're screening patients that they're not getting into the study, why aren't they getting in? And what can you do about it? Is it all just because they've said no? Or is there eligibility criteria that [they] are not* |

| | | |
|---|---|---|
| | were identified as key elements to prevent recruitment challenges. | *meeting and is there any room for movement in our eligibility criteria? Because it's too late to do that two years down the track, when you were meant to have recruited 200 participants and you've got 20." (Industry 3)* |
| | **Unique challenges in RRR health services but retention might be better than metropolitan sites.** | |
| | ▪ RRR clinical trial participants travel vast distances to take part. | Quote in text |
| | ▪ Clinical trial participation in cities by RRR patients is impractical and disruptive to care. | Quote in text |
| | ▪ Recruitment and retention of participants in RRR areas might be equivalent or better than cities. | *"We've had a lot of success in regional centers [...] it's a great place for clinical trials, patients, the participants are really pretty keen. The retention in regions is [...] certainly on par or better than recruitment in cities." (Industry 4)* |
| | ▪ Cultural factors need to be taken into consideration. | *"[Our researchers], they're very proactive with using pictures and flip charts. [...] Having that opportunity for a two-way conversation [...] taking that concern of patients seriously. [...] Which is possible mainly with the investigator led studies. With the sponsor led studies, it's much more difficult because they've [...] quite clear directions on what you have to use." (Health Service 14)* |
| **Clinical trial approval processes** | **The approval process is long and variable across Australia.** | |
| | ▪ Significant reforms underway in Australia at state and national levels that aim to improve the capacity and capability of health services to conduct high quality clinical trials. | *"National Clinical Trials Governance Framework [...] now articulates what the expectation is at a national level for hospital and health services who participate in clinical trials, what their governance and their consumer engagement responsibilities are" (Health Service 14)* |
| | ▪ Variation in research governance approval process between health services and across jurisdictions. | *"[...] Larger hospitals [in Victoria] [...] they all [...] have their own off-the-shelf product, they don't even use ERM (ethical review management online platform), they jump in and out as needed, they continue to use their own system" (Health Service 15)*<br><br>*"[...] That [RGO] didn't require a cover letter that needed a signature, but this one did. And so, it took that feedback like four weeks to get to me." (Health Service 7)* |
| | ▪ Long delays are reported throughout the research governance process. | *"Some of these things sit on their (CEO and HOD) desks for weeks. [...] These people, either through laziness, or indecision, or whatever, sit on an application." (Clinician Researcher 1)*<br><br>*"[...] [The health service lawyers] look at it (the CTRA) in about four weeks. [...] The sponsor's lawyers, they may take a few weeks as well [...] it might go back and forth. And then it'll be a couple of weeks each time." (Health Service 14)* |
| | ▪ Conflicting expectations between health service research governance and clinician researchers. | *"'There are often other delays [...] delays around the budget negotiations with the sponsor…contractual negotiations with the sponsor's legal team [...] that's not all part of the governance clock "(Health Service 17)* |
| | **Lack of clinical trial maturity is an additional challenge for RRR health services.** | |
| | ▪ Potential for ATM to support RRR health services | Quote in text |
